# Attitudes towards disclosing a mental illness: impact on quality of life and recovery

**DOI:** 10.1007/s00127-021-02081-1

**Published:** 2021-04-24

**Authors:** Lea Mayer, Patrick W. Corrigan, Daniela Eisheuer, Nathalie Oexle, Nicolas Rüsch

**Affiliations:** 1 Section of Public Mental Health, Department of Psychiatry II, University of Ulm and BKH Günzburg, Parkstr. 11, Ulm, Germany; 2 Department of Psychology, Illinois Institute of Technology, Chicago, USA

**Keywords:** Mental illness stigma, Disclosure attitudes, Quality of life, Recovery, Attitudes to disclosure questionnaire

## Abstract

**Purpose:**

The decision whether to disclose a mental illness has individual and social consequences. Secrecy may protect from stigma and discrimination while disclosure can increase social support and facilitate help-seeking. Therefore, disclosure decisions are a key reaction to stigma. The first aim of this study was to test a newly developed scale to measure disclosure attitudes, the Attitudes to Disclosure Questionnaire (AtDQ). The second aim was to examine the impact of attitudes towards disclosing a mental illness on quality of life and recovery.

**Methods:**

Among 100 participants with mental illness, disclosure attitudes, quality of life, recovery, benefits of disclosure, secrecy, social withdrawal, self-stigma, and depressive symptoms were assessed at weeks 0, 3 and 6. Psychometric properties of the AtDQ were analysed. Longitudinal associations between disclosure attitudes at baseline and quality of life and recovery after 6 weeks were examined in linear regressions.

**Results:**

The analyses of the AtDQ indicated one-factor solutions, high acceptability, high internal consistency, and good retest reliability for the total scale and the subscales as well as high construct validity of the total scale. Results provided initial support for sensitivity to change. More positive disclosure attitudes in general and in particular regarding to family at baseline predicted better quality of life and recovery after 6 weeks.

**Conclusion:**

The current study provides initial support for the AtDQ as a useful measure of disclosure attitudes. Disclosing a mental illness, especially with respect to family, may improve quality of life and recovery of people with mental illness.

**Supplementary Information:**

The online version contains supplementary material available at 10.1007/s00127-021-02081-1.

## Introduction

People with mental illness face a double problem. Besides their symptoms, many have to handle mental illness stigma. Stigma is a broad concept comprising negative stereotypes, prejudice and discrimination against people with mental illness [1]. In general, stigma has its roots in stereotypes, which are collective opinions about a social group (“People with mental illness are dangerous”). Stereotypes can lead to prejudice if people endorse them and consequently show negative emotional reactions such as anger or fear (“Yes, that is true. Therefore, I am afraid of people with mental illness”). Prejudice can result in discrimination, like hostility or avoidance, greatly reducing opportunities for stigmatised individuals within many domains (e.g. education or health care). In addition, many people who are affected by public stigma agree with stereotypes and suffer from self-stigma (“I have a mental illness, so I must be weak”) [1]. As a consequence they can experience diminished self-esteem, self-efficacy, and self-respect which may lead to feelings of hopelessness and worthlessness as well as decreased help-seeking and capability to pursue goals [2–4]. Therefore, stigma can reduce quality of life and is a major barrier to recovery [4, 5].

Since having a mental illness is often concealable, individuals face the decision whether, when and to whom to disclose to (i.e. reveal information about their mental illness). Disclosure decisions can affect social interactions, the sense of belonging, physical and psychological well-being and, therefore, are key reactions to stigma. Non-disclosure can protect from stigma and discrimination but can make it difficult to find social support or seek help and may be a burden for those remaining in the closet [6]. Chaudoir and Fisher described a model about the dynamic disclosure decision process of individuals with a concealable identity [7]. According to this model, (non-) disclosure starts with a decision-making process influenced by approach-focussed and avoidance-focussed goals resulting in a decision for or against disclosure. Disclosure can lead to several individual, dyadic, and social consequences, mediated through numerous processes [7]. Possible consequences of disclosing a mental illness are enhanced quality of life and better recovery. Corrigan and colleagues tested the link between benefits and risks of mental illness disclosure with quality of life. Benefits of being out with mental illness were associated with better quality of life in their cross sectional study [8]. In a recent longitudinal study among unemployed individuals with mental health problems, positive attitudes towards disclosing in private settings predicted better quality of life after adjusting for symptoms and socio-demographic variables [9]. In their qualitative study among individuals with mental illness Bril-Barniv and colleagues showed an association between disclosure and better recovery [10].

The current study aimed to test the link between attitudes towards mental illness disclosure and quality of life and recovery. Attitudes towards disclosure have often been measured by single items (e.g. “How comfortable would you feel to talk with a friend or family member about your mental health?” [9]). A standardised scale would be preferable but was not available to the best of our knowledge. We, therefore, developed a questionnaire to measure attitudes towards disclosing a mental illness for this trial. The first aim of the study was to test the psychometric properties of this new scale in terms of acceptability, internal consistency, retest-reliability, and sensitivity to change. We expected the scale to be positively associated with similar constructs (benefits of disclosure) and negatively associated with opposite constructs (secrecy, social withdrawal, and self-stigma).

Our second aim was to examine the impact of attitudes towards disclosure at baseline on quality of life and recovery of individuals with a mental illness after 6 weeks. We expected that positive attitudes towards disclosure would predict better quality of life and recovery (controlled for confounding variables). We further analysed these relationships with regard to four potential settings for disclosure: family, friends, work/education, and non-psychiatric healthcare professionals.

## Materials and methods

### Study design

Data were derived from a randomised controlled trial (RCT) examining the efficacy of a peer-led group programme for people with mental illness (‘Honest, Open, Proud’, HOP, formerly known as ‘Coming Out Proud’, COP, for details see [11, 12]). The RCT compared the HOP intervention, combined with treatment as usual (TAU), with a control condition of TAU alone. HOP was delivered as a manualised group intervention of three two-hour lessons over a 3-week period. The aim of HOP is to support people with their decisions regarding the disclosure of their mental illness. All outcome measures were administered at three times: at baseline (t0); 3 weeks later, or after the intervention for participants in the HOP group (t1); and at 3-week follow-up, i.e. 6 weeks after baseline (t2). The study received approval by the regional Zürich Ethics Committee (nr. 2012-0138) and was performed in accordance with the ethical standards of the 1964 Declaration of Helsinki and its later amendments. All participants provided written informed consent after being fully informed about study procedures.

### Participants

Inclusion criteria were at least one self-reported Axis I or Axis II disorder (according to DSM-IV [13]), age 18 or above, sufficient German language skills and at least a moderate level of self-reported disclosure-related distress (score 4 or higher on item “In general, how distressed or worried are you with respect to secrecy or disclosure of your mental illness to others?”, rated from 1/not at all to 7/very much). Exclusion criteria were current inpatient status, diagnosis of organic disorder, dementia or intellectual disability, and self-reported diagnosis of only a substance- or alcohol-related disorder without non-substance-related current psychiatric comorbidity (for details see [11]).

One-hundred participants completed the baseline assessment. Fifty-nine percent of the participants were female (*n* = 59). The average age was 42 years (SD = 11.3) with a range between 20 and 66 years. Two-thirds were in no current romantic relationship (*n* = 64; 64%) and nearly half were unemployed (*n* = 48; 48%; including retirement and work as homemaker). Years since first diagnosis ranged from 0 to 40 years with an average of 12 years (SD = 11.0). The most frequent mental disorders were depression (*n* = 60; 60%), psychosis (*n* = 27; 27%), and anxiety (*n* = 24; 24%; multiple answers were possible). The follow-up assessments were completed by 86 participants after 3 weeks (t1) and 87 participants after 6 weeks (t2).

### Measures

#### Attitudes to Disclosure Questionnaire

The Attitudes to Disclosure Questionnaire (AtDQ) was developed for the current study to measure attitudes towards disclosing a mental illness within different settings. Research literature about disclosing a mental illness and other concealable identities was analysed. A qualitative study in the gay and lesbian community identified five important conceptual frameworks about disclosure and its consequences (acceptance, community, comfort and happiness, shame and conformity, harm and discrimination [14]). With these results and the work done for the development of the Coming Out With Mental Illness Scale [8], a questionnaire measuring benefits and risks of disclosing a mental illness, and discussions with individuals with mental illness a pool with potential items for the AtDQ was created. Final items were selected by two of the authors (PWC; NR) in cooperation with people with mental disorders (see Appendix for the full scale). The German and English versions of the scale were developed in parallel with English–German bilingual speakers among researchers and peer group facilitators.

The questionnaire comprises four subscales with seven identical items, respectively, each subscale representing a different setting for potential disclosure (family, friends, work/education, and non-psychiatric healthcare professionals). The seven items in each subscale contain statements about the extent of disclosure, feelings while disclosing and concealing, as well as (experienced and anticipated) discrimination due to disclosure. Participants rated their level of agreement with each item from 1 to 7 (see Appendix). A total mean score as well as mean scores for each of the four subscales were calculated, with higher scores indicating more positive attitudes towards disclosing a mental illness.

#### Further measures

*Quality of life* was measured by the Manchester Short Assessment Of Quality Of Life (MANSA [15]) with 12 items covering satisfaction with life as a whole and across different life domains (e.g. “How satisfied are you with your life as a whole today?”, “How satisfied are you with your financial situation?”). Participants answered them on a 7-point Likert scale (1/couldn’t be worse, 2/displeased, 3/mostly dissatisfied, 4/mixed, 5/mostly satisfied, 6/pleased, 7/couldn’t be better). Higher mean scores from 1 to 7 suggest better quality of life (Cronbach’s *α* at baseline in this study = 0.84).

To assess *recovery,* the 24-item Recovery Assessment Scale was used (RAS [16, 17]). Each item was rated using a 5-point Likert scale from 1/strongly disagree to 5/strongly agree (e.g. “I know when to ask for help”). A higher mean score suggests better recovery (Cronbach´s *α* at baseline in this study = 0.88).

*Benefits of disclosure* were determined by the Coming Out With Mental Illness Scale (COMIS [8]), a self-report instrument to assess reasons for disclosing and concealing a mental illness. The COMIS contains 21 items, with seven items about perceived benefits of disclosing a mental illness and 14 items about reasons for keeping it a secret. Participants indicated their agreement on a 7-point Likert scale ranging from 1/strongly disagree to 7/strongly agree. The meaning of disclosure varies whether participants have already disclosed versus currently conceal their condition. Thus, the items differ depending on current status of disclosure (e.g. “I came out of the closet to be happier”, “I stay in the closet to hide my personal life”). Mean scores for benefits of disclosure and reasons for concealment for people who have disclosed and people who have not disclosed were calculated, with higher scores reflecting more perceived benefits or risks of disclosure. For our analyses, a new variable for overall benefits of disclosure was created by averaging perceived benefits of coming out for disclosers and non-disclosers (Cronbach’s *α* for overall benefits of disclosure at baseline in this study = 0.79).

*Secrecy* was measured using the 5-item Link´s Secrecy scale [18]. Participants rated the items on a 6-point Likert scale from 1/strongly disagree to 6/strongly agree (e.g. “To get a job, a former mental patient will have to hide his or her history of hospitalisation”). A mean score was calculated, with higher scores suggesting higher levels of secrecy (Cronbach’s *α* at baseline in this study = 0.74).

*Social withdrawal* was assessed by the Withdrawal subscale of Link’s Stigma Coping Orientations Scale [18]. Participants responded to each of the 7 items on a 6-point Likert scale, ranging from 1/strongly disagree to 6/strongly agree (e.g. “After being in psychiatric treatment, it's a good idea to keep what you are thinking to yourself”). A higher mean score indicates a tendency to avoid others to escape rejection due to stigma (Cronbach’s *α* at baseline in this study = 0.59).

*Self-stigma* was measured with the 29-item Internalised Stigma of Mental Illness scale (ISMI [19]). Statements were rated on a 4-point Likert scale from 1/strongly disagree to 4/strongly agree (e.g. “Stereotypes about the mentally ill apply to me”). Higher mean scores indicate more self-stigma (Cronbach’s *α* in this study at baseline = 0.92).

*Depressive symptoms* were assessed using the 15-item German version of the Centre for Epidemiologic Studies-Depression Scale (CES-D [20, 21]). Respondents indicated symptom frequency during the last week on a 4-point Likert scale (1/rarely or none of the time, 2/some or a little of the time, 3/occasionally or a moderate amount of time, 4/most or all of the time; e.g. “I felt fearful”, “I talked less than usual”). A mean score was calculated with higher scores indicating more depressive symptoms (Cronbach’s *α* at baseline in this study = 0.81).

#### Analyses

To test the psychometric properties of the AtDQ, acceptability (response rates of the items), factor structure (exploratory factor analysis), internal consistency (Cronbach’s *α*), retest reliability (intra-class correlation coefficient comparing baseline and both follow-ups of the control group), sensitivity to change (paired *t* test for baseline and follow-up after 6 weeks for HOP group participants), and construct validity (bootstrapped linear regression analysis with 1000 bootstrap replications for predicting related constructs) of the total scale and all subscales were examined. All analyses were conducted using the baseline data of all 100 participants (except retest reliability and sensitivity to change).

To test the impact of disclosure attitudes on quality of life and recovery, bivariate Pearson’s correlations between disclosure attitudes at baseline and quality of life or recovery, respectively, at t2 were calculated. In the next step, linear regression analyses tested the associations between attitudes towards disclosure at baseline (total scale and subscales) and quality of life as well as recovery at t2. For the dependent variables, we chose t2 to examine the effects of disclosure attitudes over a longer term period than at t1. Age, gender, intervention status (HOP with TAU versus TAU alone), depressive symptoms at baseline, and quality of life or recovery at baseline were included as covariates. Due to violations of the assumptions for linear regression modelling, bootstrapped regression models were used for robust confidence intervals and significance tests. All analyses were conducted using SPSS version 25.

## Results

### Psychometric properties of the AtDQ

*Acceptability.* The AtDQ was highly acceptable. The average response rate to single items at baseline was between 1.00 and 0.97 for all items.

*Factor structure.* To examine the factor structure of the total scale and the four subscales, exploratory factor analyses (EFA) with varimax rotation were completed for baseline data. For the EFA of the total scale score (across all four settings), seven new variables were calculated by averaging the four corresponding items of each subscale (family, friends, work/education, non-psychiatric healthcare professionals). The EFA yielded two factors with eigenvalues greater than 1 (Kaiser criterion) for the total scale and the subscales family, friends, and work/education. For the subscale non-psychiatric healthcare professionals there was only one factor. The eigenvalues of the second factors of the total scale and the subscales family, friends, and work/education were close to 1. As the two-factor solutions were not conceptually meaningful, we chose one-factor solutions for the total scale and all subscales in our analyses (see Online Resource for tables with factor extraction and item loadings after varimax rotation).

*Internal consistency*. Analyses of Cronbach’s alpha at baseline showed excellent to good internal consistency for the total scale and the subscales (*α* (total) = 0.92; *α* (family) = 0.81; *α *(friends) = 0.83; *α* (work/education) = 0.83; *α* (non-psychiatric healthcare professionals) = 0.87).

*Retest reliability.* Intra-class correlation coefficients (ICC) were computed to examine retest reliability as the agreement between t0, t1 and t2 within the control group. We used both follow-ups in the analysis to increase the power of the test. The total scale and the subscales indicated good to excellent retest reliability (ICC (total) = 0.79; ICC (family) = 0.91; ICC friends) = 0.92; ICC work/education) = 0.78; ICC (non-psychiatric healthcare professionals) = 0.76).

*Sensitivity to change.* We assumed that attitudes towards disclosure would improve between baseline and follow-up after 6 weeks among HOP participants. Descriptive analyses showed improved attitudes towards disclosure (total scale and subscales) from t0 to t2. Paired *t* tests yielded significant differences between baseline and t2 for items 1 (*p* = 0.023) and 3 (*p* = 0.041) of the total scale, item 8 of the subscale friends (*p* = 0.027) as well as items 1 (*p* = 0.021) and 3 (*p* = 0.002) of the subscale work/education.

*Construct validity*. Cross sectional linear regression analyses of baseline data showed a positive association of the total scale with benefits of disclosure and negative associations with secrecy, social withdrawal, and self-stigma. Analyses of the subscales showed a positive association of the subscale family with benefits of disclosure and negative associations with social withdrawal and self-stigma. The subscale friends was positively associated with benefits of disclosure and negatively associated with secrecy, social withdrawal, and self-stigma. The subscale non-psychiatric healthcare professionals were positively associated with benefits of disclosure. All analyses were controlled for age, gender, and depressive symptoms (Table 1).

**Table 1:** Linear regressions on related constructs at baseline (controlled for age, gender, and depressive symptoms)

Independent variable: AtDQ	Dependent variable	*β*	*P*	95% CI
Total	Benefits of disclosure	0.34	0.003	0.15, 0.73
	Secrecy	− 0.24	0.037	− 0.45, − 0.02
	Social withdrawal	− 0.24	0.028	− 0.39, − 0.07
	Self-stigma	− 0.26	0.006	− 0.23, − 0.07
Family	Benefits of disclosure	0.29	0.005	0.11, 0.49
	Secrecy	− 0.12	0.313	− 0.27, 0.09
	Social withdrawal	− 0.19	0.024	− 0.26, − 0.02
	Self-stigma	− 0.24	0.015	− 0.17, − 0.03
Friends	Benefits of disclosure	0.27	0.012	0.06, 0.48
	Secrecy	− 0.29	0.010	− 0.38, − 0.05
	Social withdrawal	− 0.27	0.006	− 0.29, − 0.07
	Self-stigma	− 0.23	0.003	− 0.15, − 0.04
Work/education	Benefits of disclosure	0.22	0.131	− 0.06, 0.46
	Secrecy	− 0.24	0.101	− 0.40, 0.01
	Social withdrawal	− 0.17	0.198	− 0.28, 0.05
	Self-stigma	− 0.11	0.271	− 0.11, 0.02
Non-psychiatric healthcare professionals	Benefits of disclosure	0.24	0.024	0.02, 0.40
	Secrecy	− 0.12	0.268	− 0.23, 0.07
	Social withdrawal	− 0.17	0.058	− 0.16, 0.003
	Self-stigma	− 0.19	0.051	− 0.15, 0.003

### Impact of attitudes towards disclosure on quality of life and recovery

Characteristics of the sample as well as bivariate associations of disclosure attitudes at baseline with quality of life and recovery at t2 are shown in Table 2. The number of individuals who answered the subscale work/education of the AtDQ was lower than for the other subscales. We observed moderately positive attitudes towards disclosure in the total score and for all subscales at baseline as well as of quality of life and recovery at t2. Attitudes towards disclosure in total and with respect to family, friends, and non-psychiatric healthcare professionals at baseline were positively correlated with quality of life and recovery at t2.

**Table 2:** Descriptive statistics and correlations of the AtDQ at baseline with quality of life and recovery at t2

AtDQ at t0	M (SD)	Range of observed scores [possible range]	Correlations with			
			Quality of life at t2 (*M* = 4.32; SD = 1.10)		Recovery at t2 (*M* = 3.69; SD = 0.56)	
			*r*	*p*	*r*	*p*
Total (*N* = 99)	4.28 (0.97)	1.25–6.93 [1–7]	0.52	< 0.001	0.54	< 0.001
Family (*n* = 94)	4.32 (1.20)	1.00–7.00 [1–7]	0.53	< 0.001	0.52	< 0.001
Friends (*n* = 96)	4.51 (1.26)	1.00–7.00 [1–7]	0.44	< 0.001	0.47	< 0.001
Work/Education (*n* = 81)	3.06 (1.35)	1.00–7.00 [1–7]	0.18	0.144	0.22	0.070
Non-psychiatric healthcare professionals (*n* = 95)	4.93 (1.40)	1.43–7.00 [1–7]	0.39	< 0.001	0.44	< 0.001

Attitudes towards disclosure in total and with regard to family at baseline were significantly and positively associated with quality of life and recovery at t2, controlling for age, gender, intervention status, depressive symptoms at baseline, and quality of life or recovery at baseline (models 1 and 2 in Table 3 and models 1 and 2 in Table 4). The models explained about two-thirds of the total variance of quality of life and recovery. Attitudes towards disclosure with regard to friends, work/education, and non-psychiatric healthcare professionals were not significantly associated with increased quality of life or recovery 6 weeks later (controlled for age, gender, intervention status, depressive symptoms at baseline, and quality of life or recovery at baseline).

**Table 3:** Linear regression models on quality of life at t2

Model	Independent variables at t0	*β*	*p*	95% CI	Adjusted *R*^2^
1	AtDQ total scale	0.18	0.045	0.00, 0.37	0.69
	Age	− 0.07	0.201	− 0.02, 0.01	
	Gender	0.00	0.982	− 0.28, 0.28	
	Intervention status	0.09	0.150	− 0.07, 0.47	
	Depression	0.13	0.192	− 0.10, 0.44	
	Quality of life	0.84	0.001	0.73, 1.04	
2	AtDQ family subscale	0.19	0.018	0.04, 0.30	0.66
	Age	− 0.05	0.388	− 0.02, 0.01	
	Gender	0.00	0.957	− 0.26, 0.27	
	Intervention status	0.09	0.203	− 0.10, 0.44	
	Depression	0.14	0.191	− 0.09, 0.53	
	Quality of life	0.81	0.001	0.69, 1.08	
3	AtDQ friends subscale	0.13	0.127	− 0.03, 0.24	0.67
	Age	− 0.06	0.326	− 0.02, 0.01	
	Gender	0.02	0.701	− 0.23, 0.34	
	Intervention status	0.08	0.216	− 0.10, 0.46	
	Depression	0.10	0.311	− 0.14, 0.41	
	Quality of life	0.84	0.001	0.76, 1.05	
4	AtDQ work/education subscale	0.07	0.375	− 0.05, 0.15	0.64
	Age	− 0.09	0.163	− 0.02, 0.01	
	Gender	0.01	0.839	− 0.29, 0.36	
	Intervention status	0.04	0.603	− 0.26, 0.47	
	Depression	0.11	0.422	− 0.22, 0.50	
	Quality of life	0.89	0.001	0.73, 1.18	
5	AtDQ non-psychiatric healthcare professionals subscale	0.09	0.339	− 0.06, 0.20	0.66
	Age	− 0.04	0.495	− 0.02, 0.01	
	Gender	− 0.02	0.730	− 0.35, 0.26	
	Intervention status	0.07	0.309	− 0.13, 0.44	
	Depression	0.11	0.245	− 0.13, 0.46	
	Quality of life	0.87	0.001	0.74, 1.06	

Intervention status: 1 = HOP + TAU, 0 = TAU alone

**Table 4:** Linear regression models on recovery at t2

Model	Independent variables at t0	*β*	*p*	95% CI	Adjusted *R*^2^
1	AtDQ total subscale	0.22	0.035	0.00, 0.24	0.61
	Age	− 0.11	0.106	− 0.01, 0.002	
	Gender	− 0.09	0.183	− 0.27, 0.06	
	Intervention status	0.11	0.115	− 0.02, 0.27	
	Depression	− 0.05	0.641	− 0.19, 0.11	
	Recovery	0.61	0.001	0.38, 0.87	
2	AtDQ family subscale	0.23	0.020	0.03, 0.19	0.64
	Age	− 0.08	0.165	− 0.01, 0.002	
	Gender	− 0.12	0.066	− 0.31, 0.04	
	Intervention status	0.10	0.139	− 0.05, 0.27	
	Depression	− 0.11	0.298	− 0.25, 0.06	
	Recovery	0.59	0.001	0.40, 0.79	
3	AtDQ friends subscale	0.17	0.077	− 0.003, 0.15	0.63
	Age	− 0.07	0.266	− 0.01, 0.003	
	Gender	− 0.11	0.139	− 0.28, 0.04	
	Intervention status	0.11	0.135	− 0.05, 0.29	
	Depression	− 0.15	0.106	− 0.28, 0.02	
	Recovery	0.59	0.001	0.34, 0.81	
4	AtDQ work/education subscale	0.09	0.392	− 0.04, 0.11	0.54
	Age	− 0.12	0.122	− 0.01, 0.002	
	Gender	− 0.05	0.579	− 0.30, 0.15	
	Intervention status	0.10	0.287	− 0.11, 0.32	
	Depression	− 0.09	0.473	− 0.27, 0.12	
	Recovery	0.67	0.001	0.49, 0.95	
5	AtDQ non-psychiatric healthcare professionals subscale	0.14	0.278	− 0.04, 0.15	0.59
	Age	− 0.09	0.152	− 0.01, 0.003	
	Gender	− 0.10	0.163	− 0.28, 0.06	
	Intervention status	0.11	0.145	− 0.06, 0.32	
	Depression	− 0.08	0.472	− 0.24, 0.11	
	Recovery	0.64	0.001	0.39, 0.88	

Intervention status: 1 = HOP + TAU, 0 = TAU alone

## Discussion

The first aim of the study was to test the psychometric properties of the newly developed AtDQ. Our findings indicated one-factor solutions and good psychometric properties of the total scale and its subscales. Our analyses showed different levels of retest-reliability of the total scale and the subscales. A small sample size (in this study between 30 and 45 participants per subscale) can influence the intra-class correlation coefficient leading to inaccurate estimates. Therefore, results regarding the retest-reliability should be interpreted with caution. Results about the construct validity of the subscales and the sensitivity to change of the whole scale were not consistently significant. Half of the participants were unemployed, therefore, conclusions with respect to the work/education setting are limited.

The second aim of our study was to examine the link between attitudes towards disclosing a mental illness and quality of life and recovery. Our hypothesis regarding a positive impact of attitudes towards disclosing a mental illness at baseline on quality of life and recovery 6 weeks later was partially confirmed. The results are in line with recent findings of a positive association between disclosing a mental illness, quality of life and recovery [9, 10]. People disclosing their mental illness, especially with respect to family members, may receive support as well as experience acceptance and less stigmatising responses [10, 22]. This can make a disclosure more comfortable and, therefore, may improve quality of life [8, 23]. Disclosure of a mental illness can also enhance psychological growth and help to address the need of support, hence contribute to the recovery process [10].

Our results also are consistent with the disclosure process model of Chaudoir and Fisher [7] and disclosure’s influence on individual psychological outcomes such as quality of life and recovery. According to this model, mediating processes can affect the link between disclosure and potential outcomes (e.g. the way people interact with each other after disclosing). Both positive and negative effects on disclosure outcomes are possible [7]. Further research should examine the impact of disclosing a mental illness in different social settings on quality of life and recovery in combination with potential mediating variables (e.g. social support). Disclosure decisions are a complex and personal process which cannot be fully captured with quantitative studies. Therefore, qualitative research on this topic is needed.

The more we know about processes and consequences of disclosing a mental illness, the better we can support people with mental illness with their decisions. The Honest, Open, Proud (HOP) programme is such an intervention helping people with mental illness with their disclosure decisions. In recent RCTs HOP reduced stigma-related stress, self-stigma, depressive symptoms, and improved quality of life [11, 12, 24, 25]. However, disclosure remains risky in stigmatising settings and programmes such as HOP should be accompanied by public anti-stigma interventions.

Limitations of our study need to be considered. Participants were a non-representative sample of individuals with mental illness. Although we found support for the psychometric properties of the AtDQ, retest-reliability, sensitivity to change and construct validity of the subscales need to be examined in future studies. Regarding the associations of disclosure attitudes with quality of life and recovery, the follow-up period was short.

## Conclusions

The AtDQ is a promising instrument for future research of mental illness and disclosure that deserves further evaluation. The key strength of the scale is its opportunity to measure attitudes towards disclosure across different settings. Our findings suggest that disclosing a mental illness, especially to family members, has a positive impact on quality of life and recovery. Programmes to support individuals with mental illness in their disclosure decisions in combination with public anti-stigma interventions could improve quality of life and recovery among this group.

### Supplementary Information

Below is the link to the electronic supplementary material.

Supplementary file1 (DOCX 18 KB)

## Data Availability

All data used in this manuscript will be provided by the corresponding author upon request.

## Appendix

Attitudes to Disclosure Questionnaire

(Note: German version of the AtDQ will be provided by the corresponding author upon request.)

## Attitudes to Disclosure Questionnaire (AtDQ)

### Instructions

How do you feel about disclosing your mental illness to others or about keeping it a secret? In terms of consequences of disclosure, what have you experienced in the past, and what do you expect for the future?

For many people it matters with whom they talk about their mental illness. Therefore, all our following questions about secrecy and disclosure refer to disclosing to a certain group of people, e.g. family members, one group on each page. This group is mentioned at the top of each page.

For all questions, please circle the number between 1 and 7 that reflects what you think.

### Part 1

With respect to my extended relatives and family.

### Part 2

With respect to my friends.

### Part 3

With respect to people at work, school, university or other educational settings.

### Part 4

With respect to professionals in non-psychiatric healthcare settings (family doctor, other doctors for physical illness, nurses etc.)
