# Supplementary material for: Attitudes towards disclosing a mental illness: impact on quality of life and recovery
Source: Soc Psychiatry Psychiatr Epidemiol. 2021 Apr 24;57(2):363–74. doi: 10.1007/s00127-021-02081-1 (PMC8784363; doi:10.1007/s00127-021-02081-1)
Supplement: Supplementary file 1 — Supplementary file1 (DOCX 18 KB) [file 127_2021_2081_MOESM1_ESM.docx]

**Supplementary Material**

**Attitudes towards disclosing a mental illness: Impact on quality of life and recovery**

*Social Psychiatry and Psychiatric Epidemiology*

Lea Mayer*; Patrick W. Corrigan; Daniela Eisheuer; Nathalie Oexle_;_ Nicolas Rüsch

***** Corresponding author: Lea Mayer, Section of Public Mental Health, Department of Psychiatry II, University of Ulm and BKH Günzburg, Parkstr. 11, D – 89073 Ulm, Germany; Phone: +49 731 500 62303, email: lea.mayer@uni-ulm.de; ORCID: 0000-0002-1420-6152

**Table 1.**

Factor extraction with explained variance of the AtDQ.

| **AtDQ** | **Factor** | **Eigenvalue** | **Explained variance** |
| --- | --- | --- | --- |
| Total | 1 | 3.86 | 55.09 |
|  | 2 | 1.09 | 15.62 |
| Family | 1 | 3.32 | 47.36 |
|  | 2 | 1.22 | 17.46 |
| Friends | 1 | 3.54 | 50.54 |
|  | 2 | 1.02 | 14.62 |
| Work/education | 1 | 3.66 | 52.24 |
|  | 2 | 1.16 | 16.58 |
| Non-psychiatric healthcare professionals | 1 | 3.96 | 56.52 |

Note: Only factors with eigenvalues greater than 1 are shown.

**Table 2.**

Factor structure of the AtDQ after varimax rotation.

| **AtDQ** | **Item** | **Factor 1** | **Factor 2** |
| --- | --- | --- | --- |
| Total | 1 | 0.248 | 0.810 |
|  | 2 | 0.641 | 0.630 |
|  | 3 | 0.623 | 0.595 |
|  | 4 | -0.079 | 0.737 |
|  | 5 | 0.430 | 0.609 |
|  | 6 | 0.880 | 0.257 |
|  | 7 | 0.857 | -0.031 |
| Family | 1 | 0.780 | 0.117 |
|  | 2 | 0.816 | 0.265 |
|  | 3 | 0.826 | 0.186 |
|  | 4 | 0.415 | 0.244 |
|  | 5 | 0.689 | 0.069 |
|  | 6 | 0.271 | 0.886 |
|  | 7 | 0.115 | 0.938 |
| Friends | 1 | 0.895 | -0.040 |
|  | 2 | 0.761 | 0.459 |
|  | 3 | 0.684 | 0.457 |
|  | 4 | 0.515 | 0.197 |
|  | 5 | 0.467 | 0.434 |
|  | 6 | 0.182 | 0.896 |
|  | 7 | 0.188 | 0.843 |
| Work/Education | 1 | 0.581 | 0.612 |
|  | 2 | 0.866 | 0.184 |
|  | 3 | 0.815 | 0.110 |
|  | 4 | -0.113 | 0.815 |
|  | 5 | 0.398 | 0.721 |
|  | 6 | 0.826 | 0.154 |
|  | 7 | 0.761 | 0.066 |
